# Cryo-EM structure of the *Mycobacterium smegmatis* MmpL5-AcpM complex

**DOI:** 10.1128/mbio.03035-24

**Published:** 2024-10-31

**Authors:** Rakesh Maharjan, Zhemin Zhang, Philip A. Klenotic, William D. Gregor, Georgiana E. Purdy, Edward W. Yu

**Affiliations:** 1 Department of Pharmacology, Case Western Reserve University School of Medicine, Cleveland, Ohio, USA; 2 Department of Molecular Microbiology and Immunology, Oregon Health and Science University, Portland, Oregon, USA; Dana-Farber Cancer Institute, Boston, Massachusetts, USA

**Keywords:** Mycobacterial membrane protein Large, MmpL5, acyl carrier protein M, mycobactin L, siderophore export, iron acquisition

## Abstract

*Mycobacterium tuberculosis*, the causative agent of the airborne infection tuberculosis (TB), contains 13 mycobacterial membrane protein large (MmpL) transporters that can be divided into two distinct subclasses. These MmpL proteins play important functional roles within the mycobacterium and subsequently are considered attractive drug targets to combat TB infection. Previously, we reported both X-ray and cryo-electron microscopy (cryo-EM) structures of the MmpL3 transporter, providing high-resolution structural information for this subclass of the MmpL proteins. Thus far, there is no structural information available for the other subclass, which includes MmpL5, an inner membrane transporter that plays a critical role in iron hemostasis. Here, we report the first cryo-EM structure of the *Mycobacterium smegmatis* MmpL5 transporter bound with the meromycolate extension acyl carrier protein M (AcpM) to a resolution of 2.81 Å. Our structural data reveals that MmpL5 and AcpM interact in the cytoplasm to form a complex, and this allows us to propose that MmpL5 may also associate with the mycobactin L (MbtL) protein in a similar fashion to form a heterocomplex important for iron acquisition, which enables the survival and replication of the mycobacterium.

**IMPORTANCE:**

The emergence and spread of multidrug-resistant tuberculosis (TB) present enormous challenges to the global public health. The causative agent, *Mycobacterium tuberculosis*, has now infected more than one-third of the world's population. Here, we report the first structure of the mycobacterial membrane protein large 5 (MmpL5), an essential transporter for iron acquisition, bound with the meromycolate extension acyl carrier protein M (AcpM), indicating a plausible pathway for mycobactin translocation. Our studies will ultimately inform an era in structure-guided drug design to combat TB infection.

## OBSERVATION

*Mycobacterium tuberculosis* (Mtb) is the causative agent of the airborne infection tuberculosis (TB). It has infected approximately 1/3 of the world’s population, most having the latent form of disease with 10% progressing to active TB (1). TB is one of the deadliest human pathogens, exceeding both malaria and HIV (1, 2). It is estimated that each year TB kills more than 1.3 million people worldwide. Unfortunately, the emergence and spread of multidrug-resistant TB has worsened over the years and presents enormous challenges to the global public health.

The cell envelope of Mtb represents one of the most complex membranes of all bacteria. The outer mycomembrane is very rigid and extremely impermeable, providing a strong barrier to a wide range of antimicrobials (3). The architecture of this outer layer is defined by distinctive long-chain mycolic acids, which are either covalently linked to the arabinogalactan-peptidoglycan layer as mycolyl arabinogalactan peptidoglycans or incorporated into trehalose dimycolates. In addition, the outer leaflet possesses other non-covalently associated lipids, such as phthiocerol dimycocerosates and sulfolipids to further protect the bacterium against the host immune response (3).

The genome of Mtb encodes 13 mycobacterial membrane protein large (MmpL) transporters (4). These membrane proteins belong to a subfamily of the resistance-nodulation-cell division (RND) superfamily of transporters (5). They are critical for mycobacterial physiology by facilitating the transport of fatty acids and lipid components to the mycobacterial cell envelope. The phylogenetic tree reveals that these MmpL proteins can be separated into two distinct subclasses (6). The majority of MmpLs, including MmpL4, MmpL5, MmpL7, MmpL8, and MmpL10, are affiliated with subclass I. The predicted overall protein structure of this subclass of MmpL proteins only contains a transmembrane domain and a periplasmic domain (6). However, subclass II, including MmpL3 and MmpL11, are predicted to have an additional C-terminal cytoplasmic domain (6, 7).

**Fig 1:**
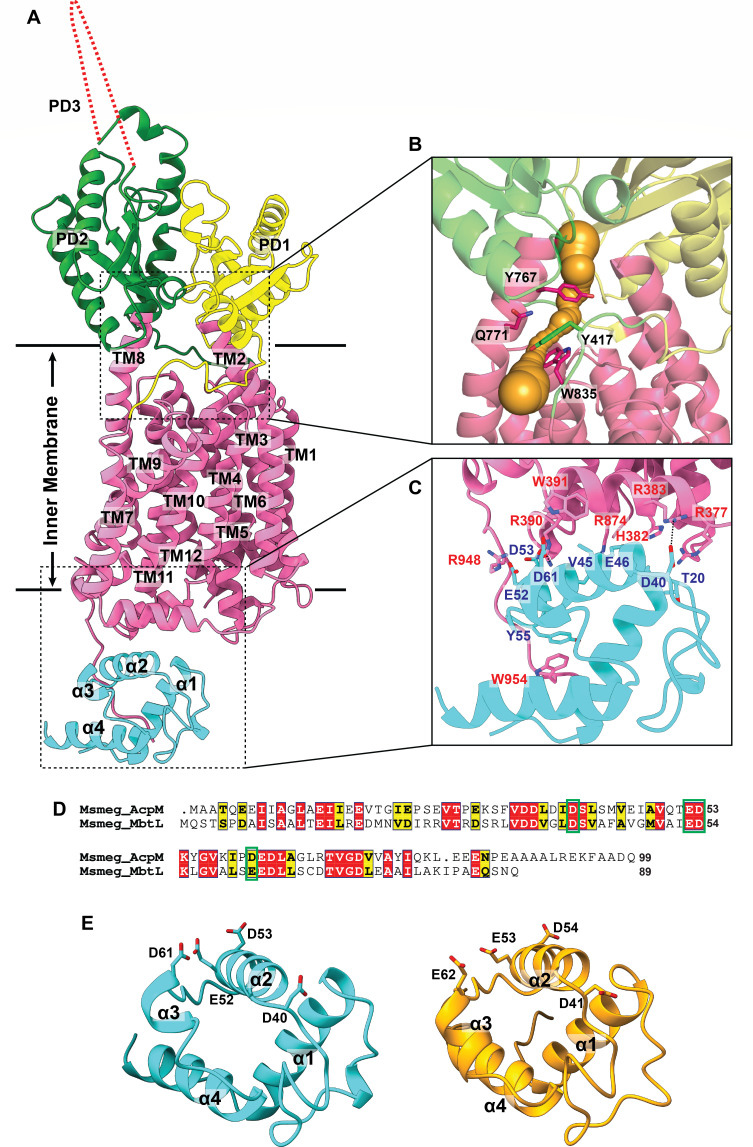
Structure of the *M. smegmatis* MmpL5-AcpM complex. (**A**) Ribbon diagram of the structure of MmpL5-AcpM viewed in the membrane plane. The secondary structural elements of the TM domain, PD1 subdomain, and PD2 subdomain of MmpL5 are colored magenta, yellow, and green, respectively. The missing PD3 subdomain is represented by a red-dotted curve. The secondary structural elements of AcpM are colored cyan. (**B**) Channel formation in the MmpL5 transporter. MmpL5 forms a channel (orange) spanning the outer leaflet of the inner membrane up to the periplasmic domain. Residues Y417, Y767, Q771, and W835 create the narrowest region of this MmpL5 channel. (**C**) Specific interactions between MmpL5 and AcpM. MmpL5 and AcpM interact with each other via hydrogen-bonding, electrostatic, and hydrophobic interactions as described. The hydrogen bonds are represented by dotted lines. (**D**) Protein sequence alignment of AcpM and MbtL. The alignment indicates that these two proteins possess 33% sequence identity. The conserved and similar residues are highlighted with red and yellow colors. The negatively charged residues of MbtL that have high identity or similarity with those of AcpM and are predicted to be important for interacting with MmpL5 are highlighted with green boxes. (**E**) Ribbon diagrams of the structure of AcpM and predicted structure of MbtL. AcpM and MbtL are colored cyan and orange, respectively. The four negatively charged residues that have high identity or similarity between these two proteins are depicted as sticks.

We previously reported high-resolution structures of the *M. smegmatis* MmpL3 transporter (8, 9), a trehalose monomycolate (TMM) transporter critical for mycobacterial cell wall biogenesis. We also determined the structures of MmpL3 bound with the substrate TMM (9). This information has allowed us to propose a mechanism for TMM lipid translocation to facilitate cell wall biosynthesis (8, 9). Additionally, we recently reported the first structural characterization of MmpL4 and MmpL5 (10), members of subclass I of MmpL transporters. MmpL5 shares similar functions with MmpL4, where they both contribute to the export of siderophores such as mycobactins (Mbts) and are essential for iron acquisition (11). To obtain a more comprehensive picture of this important MmpL family of membrane proteins, we overexpressed the *M. smegmatis* full-length MmpL5 transporter in *M. smegmatis* MC^2^-155 cells and purified it using Ni^2+^ affinity and Superdex 200 sizing columns. We then collected single-particle cryo-electron microscopy (cryo-EM) images of this protein and solved its structure to a resolution of 2.81 Å (Fig. 1; Fig. S1; Table S1). The secondary structural assignments of the cryo-EM structure are described in the supplementary information section.

MmpL5 constitutes a large membrane-spanning domain with 12 transmembrane helices (TMs 1–12) and a large periplasmic domain created by two hydrophilic loops located between TMs 1 and 2 (loop 1) and between TMs 7 and 8 (loop 2), consistent with the signature features of the resistance-nodulation-cell division (RND) superfamily of proteins (5). These two loops coordinate and contribute to create the periplasmic subdomains PD1 and PD2 (Fig. 1A). Our cryo-EM structure contains most of the MmpL5 residues, but the densities for residues 495–689, presumably forming the periplasmic subdomain PD3, are missing in the map. Overall, the cryo-EM structure of MmpL5 substantiates the prediction that this membrane protein contains only transmembrane and periplasmic domains.

Similar to the MmpL3 transporter, MmpL5 forms an elongated channel that spans the pocket surrounded by TMs 7–10 at the outer leaflet of the cytoplasmic membrane and the cavity generated between subdomains PD1 and PD2 in the periplasm (Fig. 1B). This elongated channel may form a passageway for MmpL5 to export siderophores for iron acquisition.

Surprisingly, the cryo-EM map depicts that there is an extra density attached to the MmpL5 transporter. This extra density likely represents a fortuitous protein that copurified during sample preparation. The size of this protein is quite small, where its shape can be modeled by an all α-helical bundle. Using the sequence_from_map program implemented in the PHENIX suite (12), we were able to identify this fortuitous protein as the *M. smegmatis* meromycolate extension acyl carrier protein M (AcpM), an 11-kDa enzyme that plays a central role in fatty acid and mycolic acid synthesis (13). The presence of this soluble protein in our MmpL5 sample was also confirmed by proteomics (Table S2).

Specifically, we observed that residues R383 and R390 of MmpL5 form two hydrogen bonds with residues D40 and D61 of AcpM, while residue H382 of MmpL5 creates a salt bridge with residue E46 of AcpM (Fig. 1C). Interestingly, the structure also depicts that residue R948 of MmpL5 forms two separate salt bridges with residue E52 and D53 of AcpM. In addition, residues R377, W391, and R874 of MmpL5 interact with residues T20, D61, and Q50 of AcpM via electrostatic interactions. Furthermore, residue W954 of MmpL5 stacks with residue Y55 of AcpM via aromatic-aromatic interaction. Additional contact is also found between residue W391 of MmpL5 and residue V45 of AcpM via hydrophobic interaction to further stabilize the binding (Fig. 1C). The buried surface area between MmpL5 and AcpM was calculated to be 1,080 Å^2^.

Our MmpL5-AcpM complex structure suggests that MmpL5 and AcpM coordinate with each other in the cytoplasm. However, these two proteins seem to have unrelated functions and participate in different biological pathways. MmpL5 is responsible for exporting mycobactins and is required for iron acquisition, whereas AcpM plays a critical role in the biosynthesis of mycolic acids and other fatty acids, as well as the acylation of oligosaccharides. It has been shown that AcpM is a carrier of meromycolic acid in the mycobacterial cytoplasm (14). We rationalize that a related carrier protein involved in the mycobactin synthesis pathway may specifically interact with MmpL5, similar to AcpM in the MmpL5-AcpM complex structure. Within the mycobactin synthesis pathway, mycobactin L (MbtL) is an acyl carrier protein that functions with MbtM and MbtN to incorporate a long-chain fatty acyl group to the core of mycobactin (15). The expression of MbtL is highly regulated by the presence of iron (16). We grew *M. smegmatis* MC^2^-155 cells to express MmpL5 using Luria-Bertani broth (LB), which has a relatively high concentration of iron of approximately 9.6 µM. At such iron concentration, the expression of MbtL is believed to be suppressed. Interestingly, AcpM and MbtL only share 33% protein sequence identity, but the negatively charged residues of AcpM (such as D40, E52, D53, and D61) that were found to be important for MmpL5-AcpM interactions have high identity or similarity with those of MbtL (Fig. 1D). These corresponding negatively charged residues in MbtL are D41, E53, D54, and E62. AlphaFold (17) predicts that the three-dimensional structure of MbtL is a four α-helical bundle possessing a hydrophobic internal core, similar to our determined structure of AcpM (Fig. 1E). There are a few acyl carrier proteins of similar size to AcpM in *M. smegmatis* MC^2^-155; however, these proteins have very low sequence identity with AcpM (between 19 and 24%), and their predicted structures, based on AlphaFold (17), are also very distinct. Importantly, these charged residues responsible for interacting with MmpL5 are only conserved between AcpM and MbtL.

Based on our observations, we propose that MmpL5 forms a complex with MbtL to function, where MbtL should occupy the same position as AcpM found in the MmpL5-AcpM cryo-EM structure. Given that MmpL5 and AcpM can form a complex, it raises the question of whether the functions of AcpM and MbtL are interchangeable. It is possible that the interaction network of mycobacterial cell wall biogenesis may be more complex than originally thought. Further structural, functional, and protein-protein interaction network studies are required to gain a complete understanding of this important mycobacterial system.

### Expression and purification of MmpL5

The *M. smegmatis* MmpL5 gene (MSMEG_1382) was cloned into the pBUN250 expression vector in frame with a 6×His tag at the C-terminus of MmpL5. The tagged MmpL5 protein was overproduced in *M. smegmatis* MC^2^-155. Cells were grown in 12 L of LB medium supplemented with 0.5% glycerol, 0.05% Tween-80, and 50 µg/mL kanamycin at 37°C overnight. The culture was then treated with 0.2% acetamide at 18°C. Cells were harvested after 20 h of induction. The collected bacteria were resuspended in low salt buffer (100 mM sodium phosphate [pH 7.2], 10% glycerol, 1 mM ethylenediaminetetraacetic acid [EDTA], and 1 mM phenylmethanesulfonyl fluoride [PMSF])) and disrupted with a French pressure cell. The membrane fraction was collected and washed twice with high salt buffer (20 mM sodium phosphate [pH 7.2], 2 M KCl, 10% glycerol, 1 mM EDTA, and 1 mM PMSF) and once with 20 mM HEPES-NaOH buffer (pH 7.5) containing 1 mM PMSF as described previously (18). The membrane protein was then solubilized in 1% (wt/vol) lauryl maltose neopentyl glycol (LMNG) overnight at 4°C. Insoluble material was removed by ultracentrifugation at 100,000 × *g*. The extracted protein was then purified with a Ni^2+^-affinity column. A final purification step was performed using a Superdex 200 size exclusion column loaded with buffer solution containing 20 mM Na-HEPES (pH 7.5) and 0.005% LMNG. The purity of the MmpL5 protein (> 95%) was judged using SDS-PAGE stained with Coomassie Brilliant Blue. The purified protein was then concentrated to 10 mg/mL in a buffer containing 20 mM Na-HEPES (pH 7.5) and 0.005% LMNG.

### Cryo-EM sample preparation

The purified *M. smegmatis* MmpL5 (2.5 µL of 2.0 mg/mL sample) was directly applied to glow-discharged holey carbon grids (Quantifoil Cu R1.2/1.3, 300 mesh), blotted for 10 s, and then plunge-frozen in liquid ethane using a Vitrobot (Thermo Fisher). Vitrified grids were transferred into cartridges for data collection.

### Data collection

Images were collected in super-resolution mode at 81 K magnification on a Titan Krios equipped with a K3 direct electron detector (Gatan). The physical pixel size was 1.07 Å/pixel (super-resolution of 0.535 Å/pixel). Each micrograph was exposed to a total dose of 40.12 e^−^/Å^2^ (defocus range of −0.8 to −1.5 µm), and 43 frames were captured using SerialEM (19).

### Data processing

The super-resolution image stack was aligned and binned by two using patch motion. The contrast transfer function (CTF) was estimated using patch CTF in cryoSPARC (20). Blob picking followed by 2D classification was applied to generate templates for automated template picking. Initially, 3,334,872 particles were selected after autopicking in cryoSPARC (20). Several iterative rounds of 2D classification followed by ab initio and heterogeneous 3D classification were performed to remove false picks and classes with unclear features, ice contamination, or carbon. The resulting 100,486 particles were subjected to nonuniform refinement resulting to a 2.81 Å resolution cryo-EM map of MmpL5 based on the gold-standard Fourier shell correlation (FSC 0.143).

### Model building and refinement

Model building of *M. smegmatis* MmpL5 was based on the cryo-EM map. The predicted MmpL5 structure based on the MmpL3 cryo-EM structure (PDB ID: 7K8B) (9) was used and fitted into the corresponding density maps using Chimera (21). The sequence_from_map program implemented in the PHENIX suite (12) was used to identify the *M. smegmatis* AcpM protein (MSMEG_4326), which occupied the extra density in the cryo-EM map. The subsequent procedures for model rebuilding were performed using Coot (22). Structural refinements were accomplished using the phenix.real_space_refine program (23) from the PHENIX suite (12). The final atomic model of the MmpL5-AcpM complex was evaluated using MolProbity (24). The statistics associated with data collection, 3D reconstruction, and model refinement are included in Table S1.

## Data Availability

Atomic coordinates and EM maps for MmpL5-AcpM have been deposited with PDB accession code 9DP6 and EMDB accession code EMD-47097.

## SUPPLEMENTAL MATERIAL

Figure S1 mbio.03035-24-s0001.tiff MmpL5-AcpM data processing.

Supplemental information mbio.03035-24-s0002.docx Supplemental text and captions.

Table S1 mbio.03035-24-s0003.docx MmpL5-AcpM cryo-EM data collection and refinement statistics.

Table S2 mbio.03035-24-s0004.docx Proteomics analysis of MmpL5-AcpM.

ASM does not own the copyrights to Supplemental Material that may be linked to, or accessed through, an article. The authors have granted ASM a non-exclusive, world-wide license to publish the Supplemental Material files. Please contact the corresponding author directly for reuse.
