# Supplementary figures and images for: Cryo-EM structure of the Mycobacterium smegmatis MmpL5-AcpM complex
Source: mBio. 2024 Oct 31;15(12):e03035-24. doi: 10.1128/mbio.03035-24 (PMC11633376; doi:10.1128/mbio.03035-24)

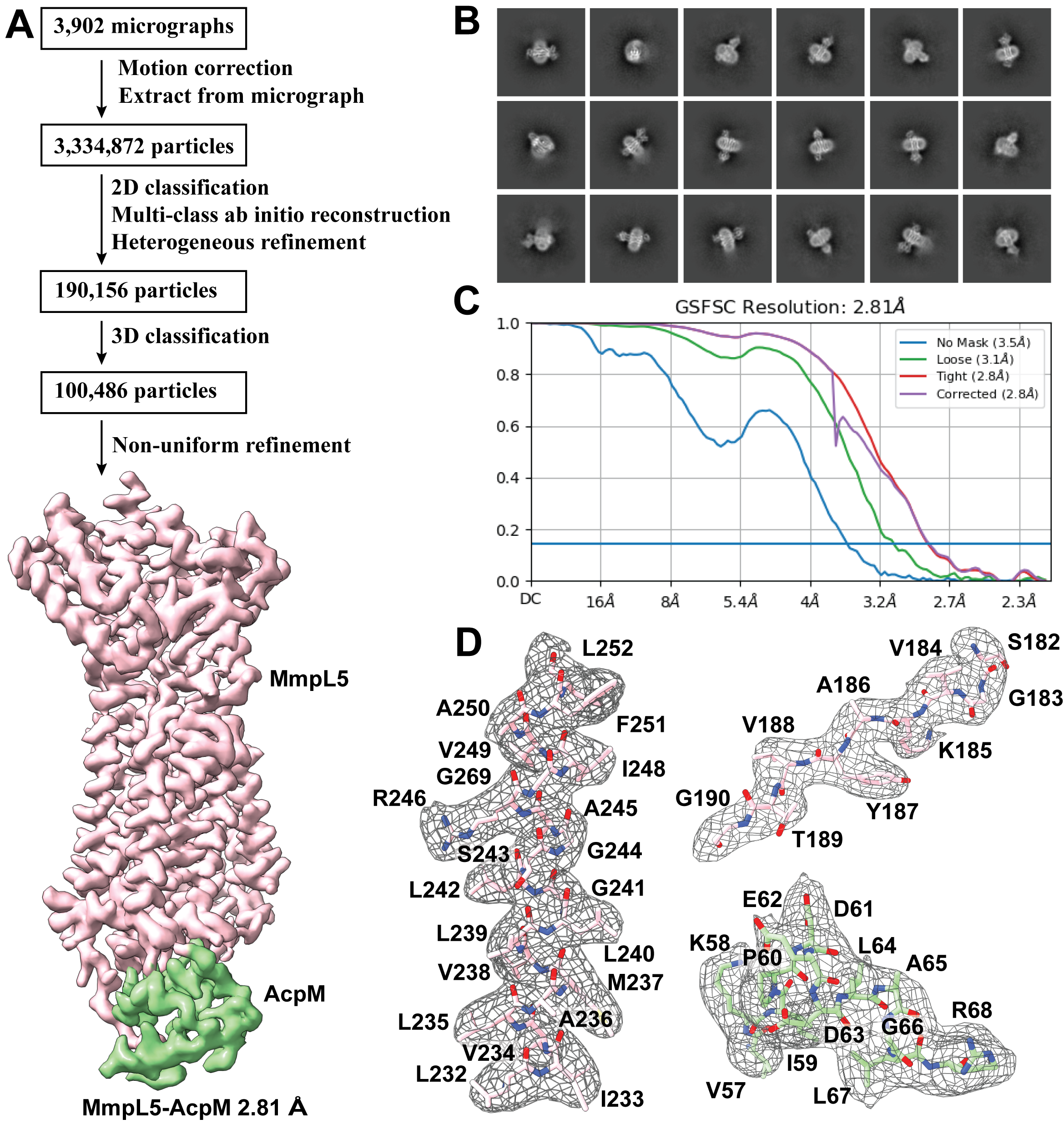

Supplement: Figure S1 — MmpL5-AcpM data processing. [file mbio.03035-24-s0001.tiff]
