# Supplementary material for: Cryo-EM structure of the Mycobacterium smegmatis MmpL5-AcpM complex
Source: mBio. 2024 Oct 31;15(12):e03035-24. doi: 10.1128/mbio.03035-24 (PMC11633376; doi:10.1128/mbio.03035-24)
Supplement: Supplemental information — Supplemental text and captions. [file mbio.03035-24-s0002.docx]

**Supplementary Information**

**Secondary structural elements of MmpL5**

The TMs, α-helices and β-strands of MmpL5 are assigned numerically from the N- to C-termini: TM1 (25-51)), α1 (51-62), α2 (72-84), β1 (91-99), α3 (105-120), β2 (126-128), α4 (138-141), β3 (148-155), α5 (162-176), β4 (184-189), TM2 (191-225), TM3 (228-255), TM4 (262-293), TM5 (297-328), TM6 (332-365), TM7 (a (379-390) and b (392-412)), α6 (420-422), α7 (428-439), α8 (442-445), β5 (448-453), α9 (461-476), β6 (481-484), α10 (695-699), α11 (701-710), β7 (717-723), α12 (731-748), β8 (757-761), TM8 (763-797), TM9 (800-826), TM10 (837-866), TM11 (868-899) and TM12 (903-935).

**Secondary structural elements of AcpM**

The α-helices of AcpM are assigned numerically from the N- to C-termini: α1 (5-19), α2 (41-54), α3 (61-67) and α4 (70-83).

**Figure S1.** MmpL5-AcpM data processing. (A) Data processing workflow of MmpL5-AcpM and side view of the MmpL5 cryo-EM map. (B) Representative 2D classes of MmpL5-AcpM. (C) Gold-Standard Fourier shell correlation (GS-FSC) curve of MmpL5-AcpM showing a resolution of 2.81 Å (FSC 0.143). (D) Representative local cryo-EM map of MmpL5-AcpM (TM of MmpL5, left; PD1 of MmpL5, middle; α3 of AcmP, right).

**Table S1.** MmpL5-AcpM cryo-EM data collection and refinement statistics.

**Table S2.** Proteomics analysis of MmpL5-AcpM.
