# Supplementary material for: Cryo-EM structure of the Mycobacterium smegmatis MmpL5-AcpM complex
Source: mBio. 2024 Oct 31;15(12):e03035-24. doi: 10.1128/mbio.03035-24 (PMC11633376; doi:10.1128/mbio.03035-24)
Supplement: Table S1 — MmpL5-AcpM cryo-EM data collection and refinement statistics. [file mbio.03035-24-s0003.docx]

| **Table S1. MmpL5-AcpM cryo-EM data collection and refinement statistics.** | |
| --- | --- |
| Data collection | MmpL5-AcpM |
| Magnification | 81,000 |
| Voltage (kV) | 300 |
| Electron Microscope | Krios-GIF-K3 |
| Defocus (um) | -0.8 to -1.5 |
| Energy filter width (eV) | 20 |
| Pixel size (Å) | 1.07 (0.535) |
| Total dose (e^-^/ Å^2^) | 40.12 |
| Number of frames | 43 |
| Number of micrographs | 3902 |
| Number of initial particles | 3,334,872 |
| Refinement |  |
| Number of total particles | 100,486 |
| GS-FSC Resolution (0.143, Å)^a^ | 2.81 |
| Model composition |  |
| Chains | 2 |
| Protein residues | 820 |
| r.m.s.d. |  |
| Bond lengths (Å) | 0.004 (0.03) |
| Bond angles (°) | 0.574 (13.66) |
| Validation |  |
| MolProbity score | 1.83 |
| Clash score | 9.38 |
| Ramachandran plot |  |
| Favored (%) | 98.28 |
| Allowed (%) | 1.72 |
| Disallowed (%) | 0.00 |
| CC Mask | 0.86 |
| ^a^Gold-Standard Fourier-Shell Correlation |  |
