# Supplementary material for: Cryo-EM structure of the Mycobacterium smegmatis MmpL5-AcpM complex
Source: mBio. 2024 Oct 31;15(12):e03035-24. doi: 10.1128/mbio.03035-24 (PMC11633376; doi:10.1128/mbio.03035-24)
Supplement: Table S2 — Proteomics analysis of MmpL5-AcpM. [file mbio.03035-24-s0004.docx]

| **Table S2. Proteomics analysis of MmpL5-AcpM.** | | | | | | | |
| --- | --- | --- | --- | --- | --- | --- | --- |
| Ranking | Protein ID | Gene  ID | Mass  kDa | Pep  unique | PSM  All | Seq.  Cov | Sequest  Score |
| **1** | **Transmembrane transport protein MmpL5** | **mmpL5** | **105.4** | **69** | **2300** | **59** | **6364.97** |
| 2 | Chaperonin GroEL 1 | groEL1 | 56.1 | 43 | 341 | 81 | 1347.61 |
| 3 | Glycerol-3-phosphate dehydrogenase | glpD2 | 60.6 | 36 | 342 | 81 | 919.14 |
| 4 | Cytochrome bc1 complex Rieske iron-sulfur subunit | qcrA | 46.3 | 33 | 318 | 72 | 724 |
| 5 | Trehalose monomycolate exporter MmpL3 | mmpL3 | 109.3 | 46 | 289 | 51 | 672.14 |
| 6 | Conserved transmembrane protein, MmpS5 | mmpS5 | 15.2 | 7 | 173 | 78 | 427.59 |
| 7 | Cyclohexanone monooxygenase | MSMEI_1000 | 56.2 | 40 | 214 | 81 | 419.88 |
| 8 | Superoxide dismutase [Cu-Zn] | sodC | 23.2 | 12 | 140 | 65 | 419.56 |
| 9 | Cytochrome bc1 complex cytochrome b subunit | qcrB | 60.2 | 26 | 215 | 53 | 401.51 |
| 10 | Isocitrate dehydrogenase [NADP] | icd2 | 82.6 | 53 | 230 | 80 | 389.43 |
| … |  |  |  |  |  |  |  |
| **21** | **Meromycolate extension acyl carrier protein** | **acpM** | **10.7** | **8** | **53** | **72** | **157.41** |
| PSM: peptide-spectrum match | | | | | | | |
